# Delivering Volumetric Hyperthermia to Head and Neck Cancer Patient-Specific Models Using an Ultrasound Spherical Random Phased Array Transducer

**DOI:** 10.3390/bioengineering12010014

**Published:** 2024-12-28

**Authors:** Muhammad Zubair, Imad Uddin, Robert Dickinson, Chris J. Diederich

**Affiliations:** 1 Department of Radiation Oncology, University of California, San Francisco, San Francisco, CA 94115, USA; 2 Department of Neurology and Neurological Sciences, Stanford University, Stanford, CA 94305, USA; 3 Department of Cardiology, Hayat Abad Medical Complex, Peshawar 23301, Pakistan; 4 Department of Bioengineering, Imperial College London, London SW7 2AZ, UK

**Keywords:** hyperthermia, drug delivery, focused ultrasound, phased array, head and neck cancer, patient-specific modeling, multifoci

## Abstract

In exploring adjuvant therapies for head and neck cancer, hyperthermia (40–45 °C) has shown efficacy in enhancing chemotherapy and radiation, as well as the delivery of liposomal drugs. Current hyperthermia treatments, however, struggle to reach large deep tumors uniformly and non-invasively. This study investigates the feasibility of delivering targeted uniform hyperthermia deep into the tissue using a non-invasive ultrasound spherical random phased array transducer. Simulations in 3D patient-specific models for thyroid and oropharyngeal cancers assessed the transducer’s proficiency. The transducer consisting of 256 elements randomly positioned on a spherical shell, operated at a frequency of 1 MHz with various phasing schemes and power modulations to analyze 40, 41, and 43 °C isothermal volumes and the penetration depth of the heating volume, along with temperature uniformity within the target area using T10, T50, and T90 temperatures, across different tumor models. Intensity distributions and volumetric temperature contours were calculated to define moderate hyperthermia boundaries. The results indicated the array’s ability to produce controlled heating volumes from 1 to 48 cm^3^ at 40 °C, 0.35 to 27 cm^3^ at 41 °C, and 0.1 to 8 cm^3^ at 43 °C. The heating depths ranged from 7 to 39 mm minimum and 52 to 59 mm maximum, measured from the skin’s inner surface. The transducer, with optimal phasing and water-cooled bolus, confined the heating to the targeted regions effectively. Multifocal sonications also improved the heating homogeneity, reducing the length-to-diameter ratio by 38% when using eight foci versus a single one. This approach shows potential for treating a range of tumors, notably deep-seated and challenging oropharyngeal cancers.

## 1. Introduction

Head and neck (H&N) cancer ranks as the sixth most commonly diagnosed cancer globally. Approximately 75% of the patients with H&N cancer are locally advanced or metastatic [1,2]. Standard treatment includes chemoradiotherapy alone or followed by a surgery, causing severe side effects especially to the organs in the head. These treatments also fail in locally advanced H&N cancer due to the recurrence at the primary site or in the lymph nodes of the neck. Up to 55% of patients will develop locoregional recurrence despite the combined treatment modality, with an overall survival of less than 60%. In recurrent H&N cancer, only 20% are eligible for curative treatment with only 26–52% of local tumor control after retreatment, resulting in a 2-year overall survival of only 10–20% after radiation and chemotherapy [3,4,5]. In addition to recurrence, severe toxicity is caused in more than 80% of patients with the current regimens, including loss of salivary function, soft tissue fibrosis, and difficulty in speaking and eating [6].

Mild hyperthermia (40–45 °C) has been recognized as a significant supplementary treatment in oncology. It has been observed to boost the effectiveness of both radiation and chemotherapy. Studies indicate that this increase is correlated with higher response rates and overall patient survival [7,8], while not inducing any toxic effects. Hyperthermia is a radiosensitizer, enhancing DNA damage and preventing repair, and can increase oxygenation in hypoxic regions of the tumor [9]. Elevating the temperature within a tumor can increase the permeability of both blood vessels and cell membranes [10], as well as improve the diffusion rate of therapeutic agents [11]. This enhancement not only diminishes interstitial pressure and alleviates hypoxia [12,13] but also improves the penetration and absorption of medications [14,15]. One of the significant breakthroughs in precise drug delivery is the development of temperature-sensitive liposomes that carry anti-cancer drugs. These liposomes are engineered to discharge their payload when exposed to locally applied heat in the hyperthermia range of 39 to 43 °C. Studies have demonstrated encouraging outcomes from the combined use of hyperthermia and these temperature-sensitive liposomes [11,16,17,18,19,20]. Doxorubicin enclosed in low temperature sensitive liposomes has also been released in high concentrations when exposed to temperatures ranging from 39.5 °C to 42.0 °C.

Numerous studies have demonstrated that hyperthermia enhances the effectiveness of chemotherapy or radiotherapy in the treatment of head and neck (H&N) cancer when used as an adjuvant therapy [21,22,23,24,25,26]; however, the technologies used to deliver hyperthermia in the previous studies were either invasive or allowed heating of only superficial regions. Moreover, there is limited spatial control of the applied thermal dose, power deposition, and volumetric heating, making it difficult to effectively heat the whole tumor. Further, the need to measure temperature with invasive means using the existing techniques is a hurdle in quantifying the temperature rise, ensuring patient safety, and avoiding thermal hotspots. Invasive temperature monitoring is limited to only few points, providing very limited spatial information and does not accurately depict the heat distribution. Recent efforts with electromagnetic arrays and accurate treatment planning have demonstrated localization of the tumor-conforming specific absorption rate (SAR) distributions as a means to improve hyperthermia to deep volumes within the neck, although the spatial limitations and control are limited to the order of centimeters due to the wavelength of the electromagnetic energy [24]. Applying local hyperthermia and restricting the temperature elevation to the volume of interest for a long period of time can be difficult with existing technologies but may uniquely be applied with high-intensity focused ultrasound under MR guidance and MR temperature monitoring [27,28,29] or US guidance with limited thermometry and treatment planning [30,31,32,33,34].

Magnetic resonance guided high-intensity focused ultrasound (HIFU) has been clinically used as a non-invasive ablative therapy for uterine fibroids [35], bone metastasis [36], essential tremor [37], and prostate cancer [38,39]. Recently, HIFU devices have also been investigated for delivering hyperthermia [29,30,31]; however, the volume heated by a HIFU transducer is usually small. Though techniques such as rapid electronic focal steering and beam forming have been used to enable large heating volumes with phased array transducer, the need to steer a single focus, or the inability to transversely steer single or multiple foci requiring mechanical transducer movement, is not practical, given the long duration of hyperthermia treatment and the need to sustain the requisite temperature increase throughout the entire tumor mass for the duration required for the drug circulation time (30–60 min). Ultrasound random phased array transducers have previously been shown to produce and steer single or multiple foci to ablate tissue with reduced grating lobes and larger steering capabilities [40,41]; however, their potential to generate and deliver volumetric hyperthermia has never been explored. Previous studies have shown the capability of such an array to generate multiple simultaneous foci and steer in both axial and transverse directions thus avoiding the need to mechanical move the transducer [42,43] and to produce 3D images useful for treatment planning and image guidance [41,44].

In this article, we investigate the optimization of an extracorporeal focused ultrasound system, based upon a spherically focused sparse array, to induce large-volume hyperthermia within neck tumors while minimizing acoustic energy and temperature elevation to surrounding tissues and structures. Three-dimensional models of both acoustics and biothermal activity were formulated to simulate the acoustic field, delineate the patterns of energy deposition, and predict the associated temperature profiles. Single or multiple foci are produced with the proper amplitude and phase distribution on each of the array element to obtain clinically relevant heating volumes with spatial uniformity. Performance metrics for evaluating the phased array transducer included the delineation of temperature contours, the extent of isothermal volumes, the breadth of temperature gradients, and the penetration depth from the surface of the skin and proximity to sensitive structures like the trachea. Customized models of head and neck (H&N) tumors and adjacent anatomical structures were constructed from segmented CT patient scans. A thorough assessment was conducted on varying potential target volumes to investigate the spectrum of clinical manifestations. The effectiveness of the phased array transducer in delivering comprehensive thermal treatment to H&N tumors, while sparing the nearby healthy tissue, was assessed.

## 2. Materials and Methods

### 2.1. The Phased Array System

**Figure 1:**
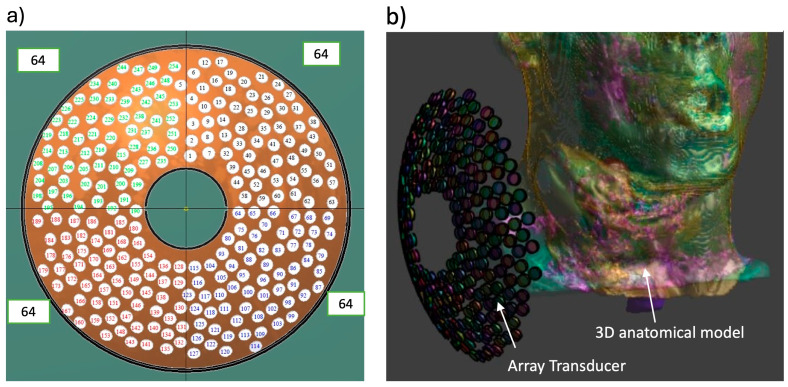
(**a**) Front face of the phased array transducer showing element distribution on the surface of the array, with four quadrants and a central hole for holding an imaging transducer, (**b**) 3D patient-specific model and bio-acoustic thermal simulation for targeted volumetric hyperthermia delivery with a random phased array transducer (1 MHz, 256 elements, 170 mm outer diameter (OD), 130 mm focal depth), positioned to target energy at a tumor in the neck. A 3D model showing the tumor and surrounding anatomy including skull bone, trachea, soft tissue, fat, and skin.

A spherical random phased array transducer consisting of 256 elements, each with 3.5 mm radius and a minimum spacing of 8 mm, sonicating at 1 MHz was used [40,41]. The elements are distributed at random across a spherical surface that measures 170 mm in diameter and has a curvature radius of 130 mm, with an F-number of 0.76. A 38 mm central hole is reserved for holding an imaging transducer for therapy guidance. Figure 1 illustrates the configuration of the element distribution for the phased array.

### 2.2. Phase Calculation

The required relative phase values needed to create single and multiple foci were determined by calculating the distances from the center of each element to the focal point. The phase and velocity distribution for each element targeting a specific focal point were computed employing the pseudoinverse technique [42,45].

(1) $$u=H^{*t}{\left(HH^{*t}\right)}^{-1}p,$$

where $u=[u_{1},u_{2},\ldots .,u_{n}]$ is the set of complex surface velocities $u_{n}$ at each $n_{th}$ element, $p=[p_{1},p_{2},\ldots .,p_{n}]$ is the set of complex pressures $p_{m}$ at each M target point, and *H* is a forward propagation operator with elements $h_{mn}=\frac{ip_{0}Z_{R}e^{i(kr_{mn}+\emptyset_{n})}}{r_{mn}}\frac{2J_{1}ka\sin\theta }{ka\sin\theta }$, where *m* and *n* are the number of control points and elements, respectively, $r_{mn}$ is the distance from the center of the $n_{th}$ element to the $m_{th}$ focus point, $\emptyset_{n}$ is the excitation phase for each element, $p_{0}$ is the pressure on the elements’ surface, a is the radius of the transducer elements, k is the wave number, $Z_{R}=ka^{2}/2$ is the Rayleigh length, and $J_{1}$ is the first-order Bessel function. $H^{*t}$ is the transpose of the complex conjugate of H. The amplitude and phase were independently regulated for each element, and a constant acoustic surface velocity and amplitude were assumed across the radiating face of each element.

### 2.3. Acoustic and Biothermal Modeling

Simulations of both the acoustic and biothermal properties were conducted using Sim4Life 6.2 (Zurich Med Tech AG, Switzerland). This software employs a finite difference time-domain solver (FDTD) for the three-dimensional linear acoustic pressure wave equation (LAPWE), with the following formulation [46].

(2) $$\rho \nabla .\frac{1}{\rho }\nabla p-\frac{1}{c^{2}}\frac{\partial^{2}p}{{\partial t}^{2}}-\frac{2\alpha }{c^{2}}\sqrt{\frac{\alpha^{2}}{\Omega^{2}}+c^{2}\frac{\partial p}{\partial t}}=0,$$

where ρ (kg/m^3^) is the density, p (Pa) is the pressure, c (m/s) is the sound speed, t (s) is the time, α (Np/m) is the absorption coefficient, and Ω (rad/s) is the angular frequency. The FDTD solver incorporates the effects of reflection, refraction, diffraction, attenuation, and interference. The three-dimensional representation of the phased array setup was created utilizing Python API within the Sim4Life modeling environment. The acoustic solver mesh’s maximum voxel dimension was determined to be one-tenth the wavelength to guarantee the precision of the simulation. It was assumed that each element’s radiating surface had a consistent acoustic surface velocity and amplitude.

To investigate the temperature distributions achievable with the phased array transducer, a 3D bioheat transfer model of patient-specific cases with Sim4Life was employed. The temperature patterns resulting from the acoustic intensity profiles were modeled using the Pennes bioheat transfer equation [47,48].

(3) $$\rho C\frac{\partial T(x,y,z)}{\partial t}=\nabla .\left[k\nabla T(x,y,z)\right]-\omega_{b}C_{b}\left[T\left(x,y,z\right)-T_{b}\right]+Q_{ac}(x,y,z),$$

where T (°C) represents the temperature of the tissue, C (J/kg/°C) signifies the specific heat capacity of the tissue, ρ (kg/m^3^) is the density of the tissue, k (W/m/°C) is the tissue thermal conductivity, $\omega_{b}$ (kg/m^3^/s) is the rate of blood perfusion, $T_{b}$ (37 °C) is the capillary blood temperature, $C_{b\;}$ (J/kg/°C) is the specific heat of blood, and $Q_{ac}$ (w/m^3^) is the acoustic power deposition in the tissue, which is derived from the acoustic pressure field, as in [49].

(4) $$Q_{ac}=\frac{{\alpha \left|p(x,y,z)\right|}^{2}}{\rho c}≅2\alpha I,$$

where *p* (Pa) represents the complex pressure, and *I* (W/m^2^) denotes the acoustic intensity. The acoustic absorption coefficient, α (Np/m), was assigned a value equal to the tissue’s attenuation coefficient, under the assumption that all acoustic energy is absorbed in situ. Dirichlet boundary conditions were established at a constant 37 °C at the peripheries of the tissue. To facilitate the interface between the transducer wall and skin, as well as to cool both the transducer and the adjacent applicator–skin/tissue interface, temperature-regulated water flows within the transducer array, which is contained by a plastic membrane. The model of convective heat loss due to the cooling flow is expressed through a convective boundary condition at the skin surface, with the heat flux defined as follows [49]:

(5) $$\hat{n}.k\left(\nabla T\right)=h(T_{cool}-T_{skin}),$$

where $\hat{n}$ is the outward normal vector, *k* represents the thermal conductivity (W/m/°C), $T_{cool\;}$ is the temperature of the cooling water flow, and $T_{skin}$ is the temperature at the skin surface. The convective heat transfer coefficient, *h*, is set at 500 W/m^2^/°C, and $T_{skin}$ is initially set to 37 °C. The cooling water circulating through the system is maintained at 20 °C. To achieve a uniform and broad temperature distribution within the tissue, with a maximum temperature of 45 °C, the output surface intensity and the phases of each transducer element were adjusted to vary the focal patterns.

### 2.4. Patient-Specific Anatomical Models

**Figure 2:**
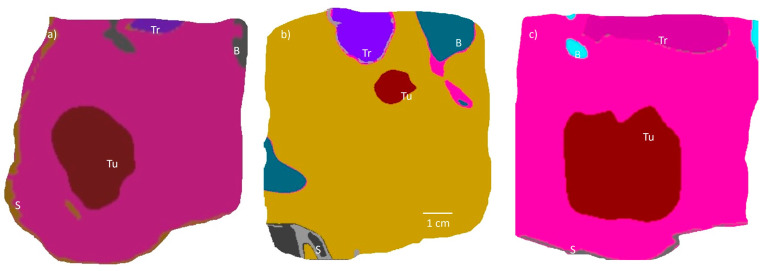
A cross-sectional plane from patient-specific models used in this study (**a**) Model 1, (**b**) Model 2, and (**c**) Model 3 showing the tumor and surrounding anatomy including bone (B), trachea (Tr), skin (S), and tumor (Tu). Models 1 and 3 are superficial tumors with varying volumes, whereas Model 2 is a deep oropharyngeal tumor.

To assess the capability of the random phased array in providing volumetric hyperthermia for the thermal treatment of head and neck tumors within a multifaceted anatomical context, custom models incorporating essential anatomical features were constructed. These three-dimensional patient-specific anatomical models were derived from CT scans of patients with neck tumors. Segmentation of these scans was performed using Mimics 19.0 (Materialize, Leuven, Belgium), creating detailed surface meshes of vital structures such as the skull, skin, soft tissue, trachea, and the tumor itself. The CT datasets were anonymized following the UCSF Institutional Review Board protocol (A18-26558 In Silico Simulation Studies with Anonymized Patient Images) and imported into the Sim4Life environment as STL files. The acoustic pressure and intensity distributions were computed using Sim4Life’s 3D acoustic solver, which operates on linear acoustic pressure wave equations and adaptive rectilinear meshes with inhomogeneous perfectly matched layer (PML) boundary conditions. A frequency of 1 MHz was applied to all transducer elements, with uniform input power to maintain a steady-state maximum temperature of 45 °C. The phase of each transducer element was tailored to the focal requirements of the target tissue. The physical attributes and perfusion rates for various tissues, such as the tumor, skull, trachea, skin, fat, and muscle, are presented in Table 1. Three case studies of neck tumors, exhibiting a wide variety in size from 1 cm^3^ to 44 cm^3^, were analyzed to validate the hyperthermia treatment approach using the phased array, with specifics provided in Table 2. Figure 2 displays two-dimensional representations of tumor layouts and adjacent anatomical structures.

## 3. Results

### 3.1. Acoustic Beam Profile

**Figure 3:**
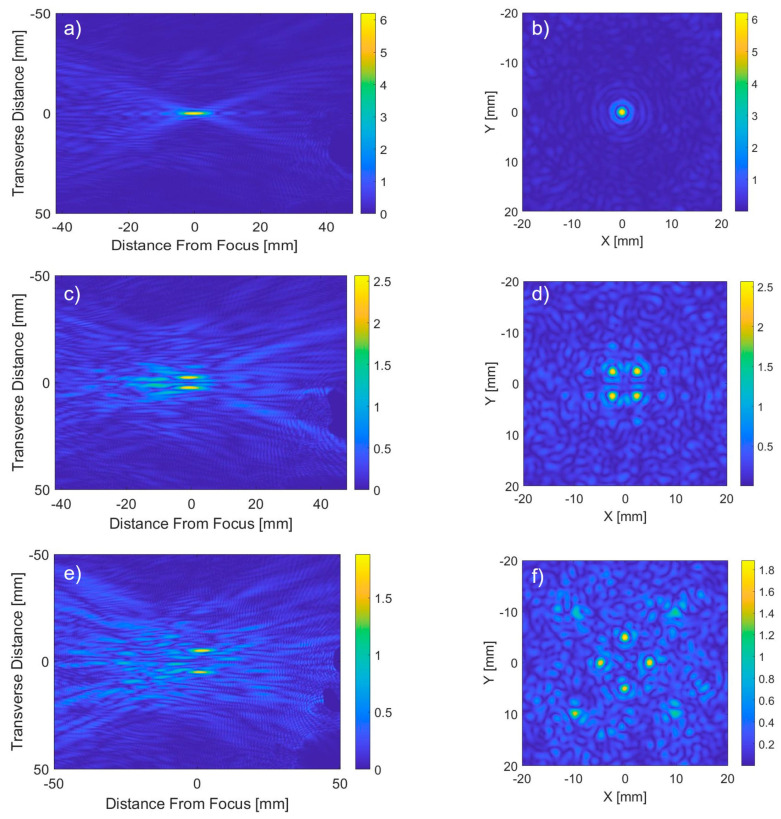
Pressure profile [MPa] projected from the phased array transducer to the patient-specific anatomical models focused at the geometric center of the array inside the tumor for a single focus in model 1 (**a**,**b**), four simultaneous foci at (x,y) = ± 2.5 mm (**c**,**d**), and eight simultaneous foci at (x,y) = ±5 mm and ±10 mm, in the axial (**a**,**c**,**e**) and transverse (**b**,**d**,**f**) planes.

Acoustic energy produced from the spherical phased array transducer propagated towards the tumor and focused on the tumor center in a heterogeneous medium is shown in Figure 3. The transducer is positioned such that the conforming water bolus covering the transducer is extended to touch the neck surface adapting its curvature. The bolus consisting of circulating water acts as a coupling and cooling medium. Longitudinal and transverse pressure distributions for single focus, four, and eight simultaneous foci are compared for a patient-specific model in Figure 3, for the same array output surface intensity of 1 W/cm^2^. It can be seen that the peak pressure is reduced by increasing the number of foci due to the redistribution of energy into multiple focal spots. The maximum pressure is reduced by 60% and 71% for four and eight foci as compared to a single focus, respectively. Some energy is reflected from the trachea contributing to the formation of standing waves, but the amplitude is too low to cause any deleterious heating.

### 3.2. Volumetric Hyperthermia in Patient-Specific Models

**Figure 4:**
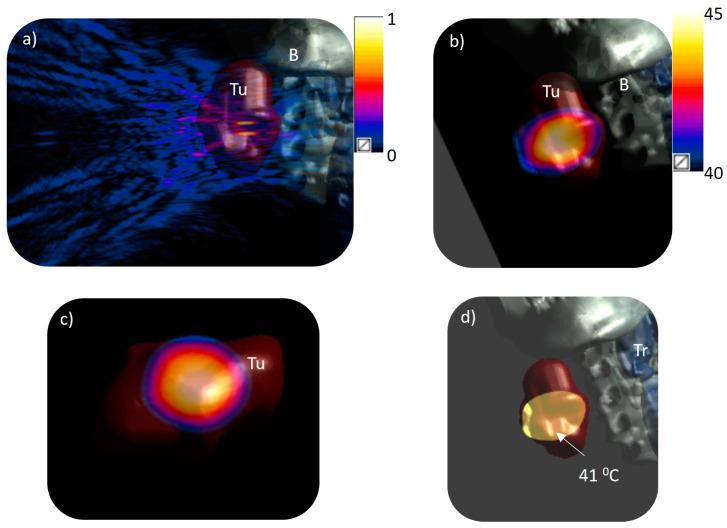
Acoustic and biothermal simulations in a 3D patient specific model (Model 1): (**a**) normalized pressure distribution projected from the phased array transducer to the model with 0.17 MPa surface pressure equivalent to 1 W/cm^2^ input surface intensity to generate four simultaneous foci (x = ±5 mm, y = ±5 mm) at the geometric center of the array (z = 130 mm); (**b**) distribution of temperature along a central axial plane; (**c**) temperature distribution across a transverse plane at the geometric center of the transducer (130 mm depth from center of transducer); (**d**) iso-temperature volume of 41 °C within the target region. Tu: tumor, B: bone, Tr: trachea.

Acoustic and thermal modeling was conducted on three patient-specific cases, using one of three selected phasing modes based on the tumor’s size in the neck region. Model 1, as depicted in Figure 2a and Table 2, features a tumor measuring 50 × 30 × 27 mm^3^ with a volume of 22 cm^3^, situated 2.5 cm beneath the inner skin surface of the neck. The 3D patient anatomy model utilized for simulation, alongside the resultant pressure and temperature profiles, is illustrated in Figure 4. At a steady state, the tumor’s temperature was raised to over 40 °C, peaking at 45 °C. The findings indicated that tumor volumes of 4.5 cm^3^ and 9 cm^3^ could be heated above 41 °C and 40 °C, respectively, using a single focus. Similarly, with four simultaneous foci, 8.9 cm^3^ and 15 cm^3^ of the tumor could be heated above 41 °C and 40 °C, respectively. The penetration depths for the heating contours 41 °C and 40 °C from the skin surface varied between 15–19 mm and 47–49 mm for a single focus and between 12–15 mm and 50–52 mm for four foci, marking the minimum and maximum extents, respectively. The width of the heating contours above 41 °C and 40 °C were 19 and 24 mm and 25 and 28 mm for single and four foci, respectively.

**Figure 5:**
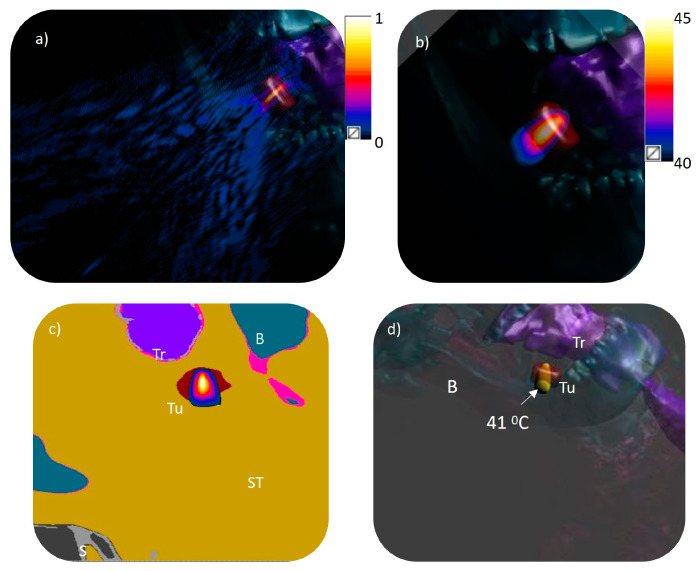
Acoustic and biothermal simulations in a 3D patient specific model (Model 2): (**a**) normalized pressure distribution projected from the phased array transducer to the model with 0.17 MPa surface pressure equivalent to 1 W/cm^2^ input surface intensity for generating a single focus at the geometric center; (**b**) distribution of temperature along a central axial plane; (**c**) temperature distribution across a transverse plane at the geometric center of the transducer (130 mm depth from center of transducer) overlaid on the voxelized image with the tumor and surrounding anatomy shown; (**d**) iso-temperature volume of 41 °C within the target region. S: skin, Tu: tumor, B: bone, Tr: trachea.

Model 2 represents a very small oropharyngeal tumor with dimensions 14 × 11 × 9 mm and 0.7 cm^3^ volume. This tumor was heated with a single focus by targeting the center of the tumor. The entire tumor was heated above 40 °C. The minimum and maximum penetration depth of 41 °C and 40 °C ranged from 42 to 57 mm and 40 to 58 mm, respectively. Figure 5 shows the pressure and temperature distributions for this patient model. It can be noted that the 40 °C contour is localized within the tumor boundaries with no heating seen in the adjacent tissues.

**Figure 6:**
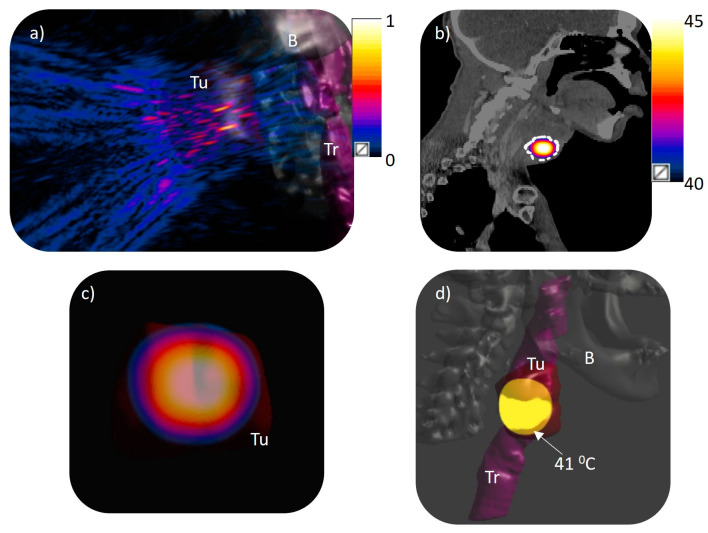
Acoustic and biothermal simulations in a 3D patient specific model (Model 3): (**a**) normalized pressure distribution projected from the phased array transducer to the model with 0.17 MPa surface pressure equivalent to 1 W/cm^2^ input surface intensity to generate eight simultaneous foci at the geometric center of the array (z = 130 mm); (**b**) temperature distribution across a central axial plane with the target delineated; (**c**) temperature distribution across a transverse plane at the geometric center of the transducer (130 mm depth from center of transducer); (**d**) iso-temperature volume of 41 °C within the target region. Tu: tumor, B: bone, Tr: trachea.

Model 3 describes a large tumor with dimensions 49 × 43 × 40 mm and volume 44 cm^3^ in the larynx region. Heating this large volume tumor with a single focus or four foci would require multiple focus scanning and both electrical and mechanical steering, significantly increasing the treatment time. The tumor was thus heated with eight simultaneous foci positioned such that the four foci in inner circle were 10 mm apart and the outer four foci in the outer circle were 20 mm apart. Figure 6 shows both the pressure and heat distribution in the patient-specific model showing the extent of the tumor being heated. It was observed that when targeted with only four foci, 13 cm^3^ and 24 cm^3^ of the tumor was heated above 41 °C and 40 °C, respectively; however, with eight foci the heated tumor volume was 27 cm^3^ and 48 cm^3^, respectively. By increasing the number of foci, the heated volume was enhanced by 100% covering the entire tumor. The simulation showed no heating on the skin, bone, or trachea. Table 3 presents a synthesis of the parameters alongside their respective acoustic performance and thermal dosimetry data.

## 4. Discussion

In this study, a simulation framework was implemented in patient-specific models of patients with H&N cancer to investigate the performance and clinical application of an ultrasound spherical random phased array transducer to deliver volumetric hyperthermia. The random phased array consists of 256 elements randomly distributed on the surface of a spherical shell that allows the beam to focus and electronically steer further off-axis and complex focal patterns with fewer side lobes or grating lobes, thus providing for dynamic beam forming and avoiding the need for mechanical movement. A frequency of 1 MHz was shown to be optimal for targeting not only superficial but also deep tumors, with limited reflections from bone or trachea due to the focused nature of the transducer. The ellipsoidal focal point produced by the transducer in 3 cm deep tissue was 8 × 1.6 × 1.6 mm, as produced by adjusting the relative phases at each element. The sharp focus resulting in a small confined heated volume is suitable for targeting small tumors in close vicinity to sensitive structures such as the trachea, skull, or blood vessels.

The limitations of current hyperthermia systems that can be applied for the neck include a nonhomogeneous heating profile, the presence of hot and cool spots, superficial and uneven heating, and a lack of noninvasive temperature monitoring and feedback control. Hyperthermia with MR-HIFU offers spatial control over temperature distribution and more confined heated volume than existing technologies; however, it also suffers from high localized pressures leading to nonuniform heating, unwanted hotspots, and mechanical effects due to the fast and sequential steering of an ultrasound beam [51]. Though continuous single-shot exposures can be employed to induce hyperthermia over large volumes, this requires rigorous mechanical movement and potential cold spots between the exposure due to perfusion [31]. Conventional clinical HIFU devices consisting of single-element transducer produce focal volumes, which are orders of magnitude smaller than the tumor volume. The use of a multi-focal heating approach with MR-HIFU provided enhanced spatial control and led to more uniform temperature distribution than the single focus strategy [52]; however, the MR-guided heating strategies have considerable associated cost and limitations of patient placement within the MRI scanner. By employing a multifoci approach with an ultrasound guided spherical phased array transducer, a uniform and homogeneous heat distribution can be obtained more efficiently. Invasive temperature probes may be placed for temperature feedback.

The successful delivery of a hyperthermia-released drug relies on accurate temperature elevations sufficient to induce the drug release from temperature-sensitive liposomes but not too high to reduce or stop local tissue perfusion thus decreasing the drug delivery. The heating distribution must also be conformal to the desired region to circumvent any inadvertent drug release and thermal damage to the surrounding anatomy and diminish patient discomfort. Given the diversity in the size, depth, and location of head and neck (H&N) tumors, a range of simulations incorporating different patient anatomies and phasing modes were conducted to demonstrate the capability of administering volumetric hyperthermia to various tumor dimensions and positions within the neck. The results showed that smaller tumors could be brought to a mild hyperthermia level by focusing solely on a single point at the transducer’s geometric center, eliminating the need for array steering (for example Model 2 shown in Figure 5). For large tumors such as Model 3, multiple simultaneous foci and/or electronic steering are required to heat the entire tumor uniformly, as shown in Figure 6. The ratio of the length to diameter was found to be smaller for the multifoci approach (1.1–1.6) than for the single focus (1.8–1.87), indicating the heating was confined to the beam path and the target region. Near and far-field heating is a major challenge that could be a cause of concern due to safety issues and would impact hyperthermia-mediated drug delivery. By applying optimal power and the adjustment of phase values on each transducer element, energy deposition outside the target region was minimized resulting in precise targeted heating. The four and eight foci generated broader but uniform and confined heating with no unwanted hotspots close to the skin, bone, or trachea.

Due to the transducer’s highly focused design (F# 0.76), low applicator acoustic powers are sufficient to achieve a high peak pressure at the target and elevate its temperature without the risk of nearfield heating. The multifoci approach offers benefit in terms of reduced peak pressure and enlarged heating volume. We have only simulated a maximum of eight simultaneous foci sufficient to cover a tumor of about 50 cm^3^. Large tumors can be sonicated with exposures of 12 or 16 foci or to increase the spacing between each focal point; however, the associated risk of increasing nearfield heating was not assessed in this study [53,54]. Nonetheless, the beam can be steered to target the unexposed tumor utilizing either or both electronic and mechanical steering of the transducer. The transducer can safely steer the beam 15 mm off the axis without generating any significant grating lobes and harmful hotspots [42,55]. A similar system for delivering volumetric hyperthermia for the liver and pancreas was developed albeit with limited electronic steering and phasing schemes [30,31].

To further evaluate the potential and efficacy of this array, both ex vivo and in vivo testing will be conducted. Experimental studies using ex vivo tissues will provide valuable information about the hyperthermia delivery, the temperature distribution, and the potential impact on surrounding tissues. In vivo testing will shed light on the feasibility, effectiveness, and safety of the hyperthermia treatment in a more realistic setting, which includes the effects of blood flow and large vessels. Real-time adjustments based on the current temperature profiles will be facilitated with a feedback control mechanism based on either invasive thermometry or possibly non-invasive MRTI, ensuring a more precise and controlled delivery of heat. It could be expected to enhance the system’s efficacy by eliminating cool and hot spots, improving homogeneous heating to therapeutic temperatures and dose throughout the entire volume, mitigating inadvertent thermal damage, and hopefully minimizing patient discomfort. The incorporation of a noninvasive temperature monitoring system can significantly enhance the treatment accuracy and safety.

## 5. Conclusions

In this study, the feasibility of generating volumetric hyperthermia distributions using a specific spherical random phased array was demonstrated in patient-specific models with bioacoustics and thermal simulations. In consideration of targeting head and neck cancers, this study has shown that temperatures between 40 °C and 45 °C can be delivered up to 6 cm deep in the neck non-invasively sustaining up to 50 cm^3^ of target tissue. Three different phasing modes were employed with the phased array transducer for hyperthermia treatment in three patient-specific head and neck tumor models. Three-dimensional acoustic and thermal simulations across all the three models studied demonstrated the feasibility of heating 1–50 cm^3^ tumor volume above 40 °C. Lower intensities were produced with a multifoci approach reducing the risk of producing unwanted mechanical effects while providing broader, homogeneous, and more confined heating volumes covering the entire extent of the tumors. The transducer is shown to be suitable for clinical applications of heating the H&N tumors non-invasively as an adjunct to chemoradiotherapy or targeted drug delivery. In future, experimental validations will be performed in head and neck phantoms and animal models to determine the efficacy of the transducer to deliver hyperthermia.

## Figures and Tables

**Table 1:** Material properties used in acoustic and biothermal modeling [50].

Tissue	Density (kg/m^3^)	Attenuation (Np/m)	Sound Speed (m/s)	Thermal Conductivity (W/m/°C)	Specific Heat (J/kg/°C)	Perfusion Rate (kg/m^3^/s)
Skin	1109	21.16	1624	0.37	3391	2.05
Tumor	1090	8.75	1588.4	0.49	3421	0.35
Bone	1908	185.4	2770.3	0.32	1313	0.3
Soft tissue	1090	7	1588.4	0.49	3421	0.7
Fat	911	4.35	1440.2	0.21	2348	0.52
Trachea	1080	0.44	1639.6	0.49	3568	0.66

**Table 2:** Anatomical characteristics of patient-specific models with head and neck tumors.

Model	Tumor Volume (cm^3^)	Tumor Dimensions (cm)	Depth of Tumor Margin from Skin (cm)	Minimum Distance of Tumor Margin from Trachea (cm)
1	22.6	5.0 × 3.0 × 2.7	2	3.4
2	0.78	1.4 × 1.1 × 0.9	5.4	0.5
3	43.8	4.9 × 4.3 × 4.0	1.7	2.6

**Table 3:** Summary of the parameters, performance metrics, and the corresponding thermal dosimetry for hyperthermia simulations of the patient-specific models (I_0_: surface intensity; D_max_: maximum penetration depth of therapeutic zone from skin; D_min_: minimum penetration depth of therapeutic zone from skin; V_43_, V41, V40: volume above 43, 41, 40 °C; L/D: length-to-diameter ratio; PHV: percent of heating volume, T_10_, T_50_, T_90_: upper 10th percentile, median, and lower 10th percentile temperature of the target area).

Model	No. of Foci	Position of Foci (x, y, z) mm	Applied Acoustic Power (W)	I_0_ (W/cm^2^)	Peak T (°C)	V_43_, V_41_, V_40_ (cm^3^)	D_max_ (43, 41, 40 °C) (mm)	D_min_ (43, 41, 40 °C) (mm)	L/D Ratio (43, 41, 40 °C)	PHV > 40 °C (%)	T_10_,T_50_, T_90_
1	4	(±5, ±5, 130)	1.064	0.018	45	2.61 8.9 15	42 49 52	20 15 12	1.2 1.36 1.42	68	43.05, 41.1, 40.2
2	1	(0, 0, 130)	1.32	0.014	45	0.1 0.35 0.89	55 57 58	46 42 39	1.8 1.87 1.82	100	41.5, 39.6, 38.9
3	4	(±5, ±5, 130)	1.34	0.0142	45	4.36 13.4 23	46 51 55	18 13 10	1.62 1.6 1.43	52	43.3, 42.2, 40.8
3	8	(±5, 0, 130), (0, ±5, 130) (±10, ±10, 130)	9.56	0.097	45	8.32 27 48	42 52 59	15 10 7	1.12 1.23 1.24	100	43, 40.5, 40

## Data Availability

Dataset available on request from authors.
